# The Flood-Related Behaviour of River Users in Australia

**DOI:** 10.1371/currents.dis.89e243413a0625941387c8b9637e291b

**Published:** 2018-06-14

**Authors:** Amy E Peden, Richard C. Franklin, Peter Leggat

**Affiliations:** College of Public Health, Medical and Veterinary Sciences, James Cook University, Townsville, Queensland, Australia; Royal Life Saving Society - Australia, Broadway, New South Wales, Australia; College of Public Health, Medical and Veterinary Sciences, James Cook University, Cairns, Queensland, Australia; College of Public Health, Medical and Veterinary Sciences, James Cook University, Townsville, Queensland, Australia; Faculty of Health Sciences, Flinders University, Adelaide, South Australia, Australia

## Abstract

**Introduction:**

Flooding is a common natural disaster affecting 77.8 million people and claiming the lives of 4,731 people globally in 2016. During times of flood, drowning is a leading cause of death. Flooding is a known risk factor for river drowning in Australia. With little known about river usage in Australia, this study aimed to examine the links between person demographics and self-reported participation in two flood-related behaviours, driving through floodwaters and swimming in a flooded river.

**Methods:**

A self-reported questionnaire was administered to adult river users at four high-risk river drowning locations; Alligator Creek, Townsville, Queensland; Murrumbidgee River, Wagga Wagga, New South Wales; Murray River, Albury, New South Wales; and Hawkesbury River, Windsor, New South Wales. Univariate and chi square analysis was undertaken with a 95% confidence interval (p<0.05). All river users surveyed, were also breathalysed to record an estimate of their blood alcohol content (BAC) on their expired breath. Results: 688 river users responded to the questionnaire; 676 (98.3%) answered the driving question and 674 (98.0%) answered the swimming in floodwaters questions. Of the respondents, 35.7% stated they had driven through floodwater and 18.7% had swum in a flooded river. Males were more likely (p<0.001) to report having undertaken both activities. Australian-born respondents were more likely to report having driven through floodwaters (p=0.006). Those aged 18-24 years old and those residing in outer regional areas were more likely (p<0.001) to have swum in a flooded river. Those who self-reported participating in both driving through floodwaters (p=0.001) and swimming in a flooded river (p<0.001) were significantly more likely to record contributory levels of alcohol (i.e. a BAC ≥0.05%) when breathalysed at the river.

**Discussion:**

Ensuring the safe movement of people during floods is difficult, particularly for those living in regional Australia, due in part to long distances travelled and reduced investment in infrastructure such as bridges. With males and females equally exposed, more effective prevention strategies must target both sexes and may include improved education on when it is safe to drive through (low depth, still water, stable road base) and when it is not (e.g. deep water, moving water and unstable road base). This study identified one in five respondents had swum in a flooded river, most commonly young people aged 18-24 years, with participants signficantly more likely to have recorded contributory levels of alcohol when breathalysed. Further research should examine the reasons behind participation in this behaviour, including the role of alcohol.

**Conclusion:**

Preventing drowning in floodwaters is an international challenge, made more difficult by people driving through or swimming in floodwaters. Strategies for driving through floodwaters should educate both males and females on when it is safe to drive through floodwaters and when it is not. Further research is required to improve knowledge of the poorly understood behaviour of swimming in flooded rivers.

## Introduction

Flooding is a common natural disaster 1, leading all other natural disasters with respect to the number of people affected and in resultant economic losses 2. The Centre for Research on the Epidemiology of Disasters (CRED) reported 164 floods claimed the lives of 4,731 people in 2016, with a further 77.8 million people affected 3. Drowning is a leading cause of death during times of flood 4, with floods estimated to have claimed the lives of over 500,000 people between 1980 and 2009 globally 5.

Rivers have been identified as a leading location for drowning internationally 5 and in Australia 6, and flooding is a known risk factor 7. Flooding results in the drowning deaths of 13 people, on average, per year in Australia 7.

Geographical remoteness (which includes isolation from major services such as medical assistance) is a risk factor for flood-related drowning in Australia 7. People in remote and very remote areas experience 80 and 229 times the risk respectively of drowning in a flood-related incident compared to major cities 7. An exploration of how to prevent drowning incidents during floods in rural and remote Australia is vital to reducing the risk and loss of life.

Driving through floodwaters is the leading activity prior to drowning in floodwaters, both in Australia 7^,^ 8 and internationally 9^, ^10. Recreational interaction with floodwaters, such as for swimming, also claims lives domestically in Australia 7^, ^11, as well as around the world 12^, ^13.

The need for systematic data collection for the prevention of loss of life during disasters has been identified, rather than data collected on an ad-hoc basis at the time of the emergency 10. To guide prevention efforts, including identifying those most at risk, this study aimed to survey river users on previous participation in two flood-related behaviours; driving through floodwaters and swimming in a flooded river.

## Methods

A self-reported survey of adult river users (18 years and older) at four river locations was conducted in January and February 2018 (summer, school holidays, wet season), namely Alligator Creek in Queensland (classified as Outer Regional) and the Murrumbidgee (Inner Regional), Murray (Inner Regional) and Hawkesbury (Major Cities) rivers in New South Wales. Alligator Creek was located in a national park (no cost to enter), whereas the other three sites were on public land. All locations had BBQ facilities, public toilets and the Hawkesbury site featured a boat ramp. All locations were previously identified as blackspots for fatal drowning.

Potential respondents were randomly approached and asked to participate. Once informed consent was obtained, respondents were asked a range of demographic and river usage questions, as well as questions about knowledge of drowning risk factors and alcohol consumption questions. All river users who completed a survey were also breathalysed, whereby their blood alcohol content (BAC) was estimated by recording the alcohol on their expired breath 14. For analysis, the results of the breathalysing were classified as BAC positive yes/no (i.e. a BAC ≥0.001%) and BAC contributory yes/no (i.e. a BAC ≥0.05%).

The focus of this study is the self-reported flood-related behaviour of river users in Australia. Respondents were asked two questions on flood-related behaviour: ‘Have you ever driven through floodwaters?’ and ‘Have you ever swum in a flooded river?’ Respondents could answer yes or no. This study forms part of a wider study on river usage 15 and alcohol consumption 6^, ^16.

The survey was administered as both paper-based forms and online through SurveyGizmo (www.surveygizmo.com) using iPads. Those surveys completed on paper were then transferred into SurveyGizmo on the same day the paper-based survey was undertaken. The final dataset was downloaded from SurveyGizmo into IBM SPSS V20 for analysis. To check accuracy of data entry, every tenth paper-based survey (n=56) was checked (by authors AEP and RCF). This resulted in the checking of 56 x 34 questions, resulting in a 0.7% error rate. These errors were corrected prior to analysis.

In SPSS, remoteness classification of the respondent’s postcode was coded using the Australian Standard Geographical Classifications (ASGC) 17. Residential postcode was coded to its remoteness classification using the Doctor Locator website (www.doctorconnect.gov.au).

Residential postcode of the respondent was also coded to the Index of Relative Socio-economic Advantage and Disadvantage (IRSAD) 18. The Index is ranked from 1-10, with a low score indicating relatively greater disadvantage (e.g. many people with low incomes and many people in unskilled occupations), compared to a high score which indicates a relative lack of disadvantage. For ease of analysis, IRSAD was categorised as low (rank 1-3), high (rank 8-10) and other/unknown.

Univariate analysis was undertaken as was chi square analysis with a 95% confidence interval (p<0.05). Chi square analysis was run using yes or no for each flood-related behaviour. Chi square analysis excluded the unknown variable.

Ethics approval for this study was granted by the James Cook University Human Research Ethics Committee (HREC – H7249).

## Results

**Figure 1: Two flood-related behaviours by sex of river users surveyed:**
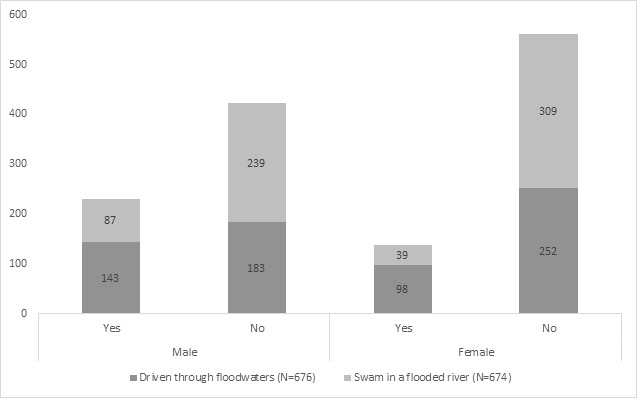

Of the 688 people surveyed, 98.3% (n=676) answered the question about driving through floodwaters and 98.0% (n=674) answered the swimming in a flooded river question. There were 35.7% of respondents who had driven through floodwaters. Males (43.9%) were more likely to have driven through floodwaters than females (27.8%) (X^2^=19.0; p<0.001) (Figure 1).

People aged 75+ years (42.9%), 65-74 year olds (40.7%) reported the highest proportion of respondents, who had driven through floodwaters; however age group did not impact likelihood of having driven through floodwaters. (Table 1)

**Table 1: Driven through floodwaters yes/no by demographic variables, chi square analysis (p value) (N=676):** Please note the unknown variable was excluded from chi square analysis

	Total		Driven through floodwaters - yes		Driven through floodwaters - no		X^2^ (p value)
	N	%	N	%	N	%	
Total	676	100.0	241	35.7	435	64.3	-
Sex							
Male	326	48.1	143	43.9	183	56.1	18.969 (p<0.001)
Female	350	51.9	98	27.8	254	72.2	
Age group							
18-24 years	190	28.1	67	35.3	123	64.7	0.017 (p=0.895)
25-34 years	144	21.3	55	38.2	89	61.8	0.516 (p=0.473)
35-44 years	124	18.3	44	35.5	80	64.5	0.002 (p=0.966)
45-54 years	120	17.8	38	31.7	82	68.3	1.010 (p=0.315)
55-64 years	64	9.5	23	35.9	41	64.1	0.003 (p=0.960)
65-74 years	27	4.0	11	40.7	16	59.3	0.318 (p=0.573)
75+ years	7	1.0	3	42.9	4	57.1	0.160 (p=0.689)
Country of birth							
Australia	571	84.5	216	37.8	355	62.2	7.598 (p=0.006)
Outside of Australia	105	15.5	25	23.8	80	76.2	
Remoteness classification of residential postcode							
Major Cities	123	18.2	34	27.9	89	73.0	4.906 (p=0.027)
Inner Regional	388	57.4	143	36.9	245	63.1	0.130 (p=0.718)
Outer Regional	136	20.1	58	42.6	78	57.4	2.999 (p=0.083)
Remote and Very Remote	6	0.9	2	33.3	4	66.7	0.023 (p=0.880)
Unknown	23	3.4	4	17.4	19	82.6	-
IRSAD classification of residential postcode							
Low	117	17.3	42	35.9	75	64.1	0.877 (p=0.349)
High	113	16.7	34	30.1	79	69.9	
Other/Unknown	446	66.0	165	37.0	281	63.0	-
Alcohol contributory (BAC ≥0.05%)							
Yes	49	7.2	28	57.1	21	42.9	10.855 (p=0.001)
No	627	92.8	213	34.0	414	66.0	

Respondents born in Australia were significantly more likely to have driven through floodwaters (37.8% yes; X^2^=7.6; p=0.006). Respondents residing in outer regional areas had the highest proportion of people driving through floodwaters (42.6%) compared to major cities (27.9%), with residents of major cities significantly less likely to have performed the behaviour (X^2^=4.9; p=0.027). Respondents classified as residing in low IRSAD areas reported a slightly higher proportion of respondents having driven through floodwaters (low 35.9%; high 30.1%). (Table 1)

Nineteen percent (19.2%) of those who self-reported having driven through floodwaters recorded a positive BAC reading, with 60.9% of those recording a BAC at contributory levels. Those who had driven through floodwaters were significantly more likely to record a BAC at contributory levels (X^2^=10.9; p=0.001). (Table 1)

Of all respondents to the swimming in a flooded river question, 18.7% stated they had swum in a flooded river. Males were significantly more likely to have swum in a flooded river (X^2^=26.5; p<0.001). Respondents aged 18-24 years were significantly more likely to self-report having ever swum in a flooded river (X^2^=17.9; p<0.001), while 45-54 year olds were significantly less likely to report having done so (X^2^=12.0; p=0.001). (Table 2)

**Table 2: Swum in a flooded river yes/no by demographic variables, chi square analysis (p value) (N=674):** Please note the unknown variable was excluded from chi square analysis.

	Total		Swum in a flooded river - yes		Swum in a flooded river - no		X^2^ (p value)
	N	%	N	%	N	%	
Total	674	100.0	126	18.7	548	81.3	-
Sex							
Male	326	48.4	87	26.7	239	73.3	26.537 (p<0.001)
Female	348	51.6	39	11.2	309	88.8	
Age group							
18-24 years	191	28.3	55	28.8	136	71.2	17.893 (<0.001)
25-34 years	143	21.2	33	23.1	110	76.9	2.294 (p=0.13)
35-44 years	124	18.4	18	14.5	106	85.5	1.745 (p=0.186)
45-54 years	120	17.8	9	7.5	111	92.5	12.036 (p=0.001)
55-64 years	63	9.3	6	9.5	57	90.5	3.845 (p=0.050)
65-74 years	26	3.9	2	7.7	24	92.3	2.154 (p=0.142)
75+ years	7	1.0	3	42.9	4	57.1	2.717 (p=0.099)
Country of birth							
Australia	571	84.7	113	19.8	458	80.2	2.950 (p=0.086)
Outside of Australia	103	15.3	13	12.6	90	87.4	
Remoteness classification of residential postcode							
Major Cities	122	18.1	21	17.2	101	82.8	0.084 (p=0.772)
Inner Regional	388	57.6	54	13.9	334	86.1	11.462 (p=0.001)
Outer Regional	136	20.2	43	31.6	93	68.4	21.086 (p<0.001)
Remote and Very Remote	5	0.7	0	0.0	5	100.0	1.116 (p=0.291)
Unknown	23	3.4	8	34.8	15	65.2	-
IRSAD classification of residential postcode							
Low	115	17.1	20	17.4	95	82.6	0.448 (p=0.503)
High	113	16.8	16	14.2	97	85.8	
Unknown	446	66.2	90	20.2	356	79.8	-
Alcohol contributory (BAC ≥0.05%)							
Yes	49	7.3	19	38.8	30	61.2	13.913 (p<0.001)
No	625	92.7	107	17.1	518	82.9	

Inner regional dwelling respondents were significantly less likely to have swum in a flooded river (X^2^=11.5; p=0.001); whereas those residing in outer regional areas were significantly more likely to have done so (X^2^=21.1; p<0.001). Country of birth and IRSAD did not significantly impact likelihood of having swum in a flooded river. (Table 2)

Twenty-two percent (22.2%) of those who self-reported ever swimming in a flooded river recorded positive BAC readings when breathalysed. Of these, 67.9% recorded BACs at contributory levels. There was a statistically significant link between those who reported having swum in a flooded river and both positive BACs (X^2^=4.4; p=0.037) and BACs at contributory levels (X^2^=13.9;p<0.001). (Table 2)

## Discussion

Flooding is one of the most deadly, and costly, of all natural disasters 2,3, the frequency of which is likely to increase due to climate change 19. Minimising the impact of such disasters, including people's interaction with floodwaters, will reduce loss of life. This study found that 36% of river users surveyed had driven through floodwaters and 19% had swum in a flooded river. Both activities were more common among males, with 18-24 year olds and people residing in outer regional areas significantly more likely to report having swum in a flooded river. There was a statistically significant link found between respondents who self-reported having participated in both risk flood-related behaviours and recording BACs at contributory levels when breathalysed at the river.

The movement of people during floods is a challenge for those living in rural Australia. Previous research 20,21,22,23 exploring factors impacting driving through and avoiding driving through floodwaters, has highlighted the issue of fatigue, a particularly important factor as an alternate route can add significant time to a journey and thus tempt drivers to cross flooded roads 20. Reduced investment in infrastructure such as bridges in regional and remote areas 24 may also contribute to an increased need to drive through floodwaters.

Simply discouraging people from driving through floodwaters is unlikely to be practical in rural Australia, particularly in areas with regular low-level flooding. More effective prevention strategies may include improved education on when it is safe to drive through (low depth, still water, stable road base) and when it is not (e.g. deep water, moving water and unstable road base). However there are challenges in identifying a stable road base and current prevention messages take a didactic approach advising “If it’s flooded, forget it” (http://floodwatersafety.initiatives.qld.gov.au/) and not to drive through.

Outer regional residents were found to have the highest proportion of respondents who self-reported having ever driven through floodwaters, as well as being significantly more likely to have previously swum in a flooded river. This may be due to the lack of infrastructure, lower initial awareness of the risk or over-familiarity with flooding leading to an underestimation of the risk. The link between participation in risky flood-related behaviours and outer regional residents requires further investigation.

Internationally, males are overrepresented in drowning statistics 5, accounting for 80% of fatal drownings overall, and fatal river drowning in Australia 6. Males have been identified as having poorer swimming skills 25 and lower levels of water safety knowledge than their female peers 26, as well as being more prone to risk-taking behaviour 27^, ^28. However, this proportion is higher than the proportion of 60% male for flood-related fatalities due to driving through floodwaters 7, although it reflects the number of people reporting in this study (i.e. the 59% of male respondents to this survey who reported having driven through floodwaters). Thus highlighting that the risk is about exposure (i.e. driving through floodwaters) rather than related to the sex of the person who drowns. While different messaging for each sex may be appropriate for the effective delivery of prevention messages, there is a need for strategies to mitigate the likelihood of people driving through floodwaters targeted at flood-prone locations, regardless of gender.

Although age was not found to be a statistically significant indicator of likelihood of having driven through floodwaters, respondents in the oldest age groups recorded the highest proportion of respondents who had undertaken the activity, with 43% of 75+ year olds and 41% of 65-74 year olds self-reporting having driven through floodwaters. As the questionnaire did not define a timeframe within which to have performed the activity (i.e. had the respondent ever driven through floodwaters), this may reflect a relatively greater number of flood seasons through which the respondent has lived and therefore, had the opportunity to drive through floodwaters, rather than any riskier behaviour being undertaken by the older age group. Further research may be warranted exploring attitudes towards driving through floodwaters among the older age group.

This study identified one in five respondents had swum in a flooded river. Unlike driving through floodwaters where as people aged, the likelihood of driving through floodwater increased (greater chance of encountering floodwater), young people (18-24 years) were more likely to report swimming in a flooded river. This dichotomy may suggest an element of risk-taking in youth, however this appears to be a recent activity, as older people were less likely to report swimming in floodwaters. Swimming in floodwaters is a poorly understood behaviour with little previous research. The survey tool did not examine the context within which the respondent had swum in a flooded river (e.g. out of necessity, skylarking or performing a rescue). Further research should examine the reasons behind this behaviour. With those residing in outer regional areas found to be more likely to have swum in a flooded river, prevention strategies must take into account the regional and remote context 29.

Alcohol is a known risk factor for drowning and aquatic-related injury 30. This study identified a statistically significant link between alcohol consumption, in particular respondents recording BACs at contributory levels, and self-reported participation in both risky flood-related behaviours being analysed. While the survey questionnaire did not ask if the respondent was under the influence of alcohol at the time of participating in these risky flood-related behaviours, it may be that alcohol contributes to a person’s decision to take risks in and around floodwaters. This is worthy of further research to better understand the motivations underlying a person’s decision to interact with floodwaters in such a way. Such information will add a helpful layer to the development of preventative messaging and campaigns 23.

As with all self-reported surveys there are limitations. These include recall bias, the survey being administered in English and the survey not defining what was meant by floodwaters (for driving through) or a flooded river (for swimming). Respondent were asked if they had ‘ever’ undertaken the two flood-related behaviours, and as such caution should be used when interpreting the age group analysis as the age at which the behaviours were performed was not captured. This study did not examine frequency of the behaviours undertaken. This was a cross-sectional study and does not determine cause and effect. The sample was a random convenience sample and therefore results represent the views of those attending the four river locations only. The survey was administered in the summer and wet season months and may impact recall. Refusals were not recorded.

## Conclusion

Preventing drowning in floodwaters is an international challenge, made more difficult by people driving through, or swimming in, floodwaters. Practical strategies to reduce loss of life due to driving through floodwaters are required, including skills to assess the risk and make informed decisions on when it is safe to drive through and when it is not. Swimming in floodwaters is a little researched topic. While this study has identified one in five people have undertaken the behaviour, commonly at a young age, there is a need for further research to understand the context of the behaviour and the motivations for engaging in it, including the role of alcohol. Such knowledge would allow for effective, regionally-specific drowning prevention strategies to be developed, targeting those most at-risk, in order to reduce loss of life during times of flood.

## Data Availability Statement

Due to ethical constraints imposed by the Ethics Committee that granted approval for this study, the data is unable to be publicly uploaded. Data requests can be made by contacting ethics@jcu.edu.au and quoting the ethics approval number H7249.

## Competing Interests Statement

The authors have declared that no competing interests exist.

## Corresponding Author

Amy Peden, Royal Life Saving Society - Australia and James Cook University (apeden@rlssa.org.au)

## References

[1] Doocy S, Daniels A, Murray S, Kirsch TD. The Human Impact of Floods: a Historical Review of Events 1980-2009 and Systematic Literature Review. PLOS Currents Disasters. 2013 Apr 16 . Edition 1. 10.1371/currents.dis.f4deb457904936b07c09daa98ee8171a PMC3644291 23857425

[2] United Nations Office for Disaster Risk Reduction (UNISDR). Guidelines for reducing flood losses. 2002.

[3] Guha-Sapir D HP, Wallemacq P. Below. R. . Annual Disaster Statistical Review 2016: The Numbers and Trends. . Brussels: Centre for Research on the Epidemiology of Disasters (CRED); 2016

[4] Di Mauro M DBK, Meloni M. Quantitative methods for estimating flood fatalities: towards the introduction of loss-of-life estimation in the assessment of flood risk. . Natural Hazards. 2012;63:1083-113.

[5] World Health Organization. Global Report on Drowning: Preventing a Leading Killer Geneva: World Health Organization; 2014.

[6] Peden A, Franklin, RC., Leggat, PA. The Hidden Tragedy of Rivers: A decade of unintentional fatal drowning in Australia. PLoS ONE. 2016;11(8):e0160709. 10.1371/journal.pone.0160709 PMC4982636 27517313

[7] Peden A, Franklin, RC., Leggat, PA., Aitken, P. Causal Pathways of Flood Related River Drowning Deaths in Australia. PLOS Currents Disasters. 2017;May 18(Edition 1).

[8] FitzGerald G, Du W, Jamal A, Clarke M, Hou J. Flood fatalities in contemporary Australia (1997–2008). Emergency Medicine Australasia. 2010;22(2):183-9. 10.1111/j.1742-6723.2010.01284.x 20534054

[9] Diakakis M, Deligiannakis G. Vehicle-related flood fatalities in Greece. Environmental Hazards. 2013;12(3-4):278-90.

[10] Jonkman SN, Kelman I. An analysis of the causes and circumstances of flood disaster deaths. Disasters. 2005;29(1):75-97. 10.1111/j.0361-3666.2005.00275.x 15720382

[11] Haynes K, Coates, L., van den Honert, R., et al. Exploring the circumstances surrounding flood fatalities in Australia - 1900-2015 and the implications for policy and practice. Environmental Science & Policy. 2017;76:165-76.

[12] French J, Ing R, Von Allmen S, Wood R. Mortality from flash floods: a review of national weather service reports, 1969-81. Public Health Reports. 1983;98(6):584-8. PMC1424497 6419273

[13] Aceto L, Aurora Pasqua, A., Petrucci, O. Effects of damaging hydrogeological events on people throughout 15 years in a Mediterranean region. Advances in Geosciences. 2017;44:67-77.

[14] Cherpitel CJ. Injury and the Role of Alcohol: County-Wide Emergency Room Data. Alcoholism: Clinical and Experimental Research. 1994;18(3):679-84. 10.1111/j.1530-0277.1994.tb00930.x 7943675

[15] Peden AE, Franklin RC, Leggat PA Exploring visitation at rivers to understand drowning riskInjury Prevention Published Online First: 06 June 2018. doi: 10.1136/injuryprev-2018-042819 10.1136/injuryprev-2018-042819 29875291

[16] Peden A, Franklin, RC., Leggat, PA. Alcohol and its contributory role in fatal drowning in Australian rivers, 2002-2012. Accident Analysis and Prevention. 2017;98:259-65. 10.1016/j.aap.2016.10.009 27771578

[17] Australian Bureau of Statistics. Statistical Geography Volume 1 - Australian Standard Geographical Classification (ASGC). Canberra: Australian Bureau of Statistics; 2006. Contract No.: 12160.

[18] Australian Bureau of Statistics. 2033.0.55.001 - Census of Population and Housing: Socio-Economic Indexes for Areas (SEIFA), Australia, 2011: Australian Government; 2013 [Available from: http://www.abs.gov.au/ausstats/abs@.nsf/Lookup/2033.0.55.001main+features100042011].

[19] Roy B, Sonia A, Luke B, Enamul H. Socioeconomic Vulnerability and Adaptation to Environmental Risk: A Case Study of Climate Change and Flooding in Bangladesh. Risk Analysis. 2007;27(2):313-26. 10.1111/j.1539-6924.2007.00884.x 17511700

[20] Hamilton K, Peden, A.E., Pearson, M., & Hagger, M.S. Stop there's water on the road! Identifying key beliefs guiding people’s willingness to drive through flooded waterways. Safety Science. 2016;86:308-14.

[21] Hamilton K, Price, S., Keech, JJ., Peden, AE., Hagger, MS. Drivers' experiences during floods: investigating the psychological influences underpinning decisions to avoid driving through floodwater. International Journal of Disaster Risk Reduction. 2018; 28: 507-518.doi.org/10.1016/j.ijdrr.2017.12.013.

[22] Franklin R, King, JC., Aitken, P.J., Leggat, PA. "Washed away" - assessing community perceptions of flooding and prevention strategies: a North Queensland example. Natural Hazards. 2014;71.

[23] Hamilton K, Peden, AE., Keech, JJ., Hagger, MS. Changing people's attitudes and beliefs toward driving through floodwaters: Evaluation of a video infographic. Transportation Research Part F. 2018;53:50-60.

[24] McKenzie F. Population Decline in Non-Metropolitan Australia: Impacts and Policy Implications. Urban Policy and Research. 1994;12(4):253-63.

[25] Franklin RC, Peden AE, Hodges S, Lloyd N, Larsen P, O’Connor C, Scarr J. Learning to Swim - What influences success? International Journal of Aquatic Research and Education. 2015;9(3):220-40.

[26] Peden AE, Franklin, RC., Scarr, J. Measuring Australian Children's Water Safety Knowledge: The National Water Safety Quiz. International Journal of Aquatic Research and Education. 2017;10(2).

[27] Hamilton, K., Schmidt, HJ. (2013). "Drinking and swimming: Investigating young Australian males' intentions to engage in recreational swimming while under the influence of alcohol." Journal of Community Health 39(1): 139-147. 10.1007/s10900-013-9751-4 23979669

[28] Moran K. (Young) Men behaving badly; dangerous masculinities and risk of drowning in aquatic leisure activities. Annals of Leisure Research. 2011;14(2-3):260-72.

[29] Beattie N, Shaw P, Larson A. Water safety in the bush: strategies for addressing training needs in remote areas. Rural and Remote Health. 2008;8(2):855. 18498202

[30] Hamilton K, Keech,, JJ., Peden, AE., Hagger, MS. (2018) Alcohol use, aquatic injury, and unintentional drowning: A systematic literature review. Drug and Alcohol Review. doi.org/10.1111/dar.12817 10.1111/dar.12817 29862582

